# Periostin regulates osteogenesis of mesenchymal stem cells from ovariectomized rats through actions on the ILK/Akt/GSK-3β Axis

**DOI:** 10.1590/1678-4685-GMB-2020-0461

**Published:** 2021-09-29

**Authors:** Silin Liu, Zuolin Jin, Meng Cao, Dandan Hao, Chunrong Li, Doudou Li, Weiwei Zhou

**Affiliations:** 1 The Fourth Military Medical University, School of Stomatology, Department of Orthodontics, State Key Laboratory of Military Stomatology & National Clinical Research Center for Oral Diseases & Shaanxi Clinical Research Center for Oral Diseases, Xi’an, China.; 2 Affiliated Hospital of Chifeng University, Department of Orthodontics, Inner Mongolia, China.; 3 Chifeng University, Medical College, Department of Physiology, Inner Mongolia, China.

**Keywords:** Osteoporosis, periostin, osteoblast, bone marrow skeletal stem cells, osteogenesis

## Abstract

Osteoporosis is a condition of the skeleton that mainly results from estrogen deficiency. Periostin is a matricellular component in bone that is involved in osteoblast differentiation. However, how Periostin promotes osteogenesis remains largely unknown. Here, we isolated bone marrow skeletal stem cells (BMSCs) derived from an ovariectomy (OVX)-induced osteoporosis rat model and the effects of periostin on BMSCs derived from OVX rats (OVX-BMSCs) were assessed. Overexpression of periostin enhanced alkaline phosphatase (ALP) and alizarin red staining in OVX-BMSCs as well as the osteogenic genes OCN, BSP and Runx2. ILK is a downstream effector of signals from the extracellular matrix and participates in bone homeostasis. Overexpression of periostin also increased expression of protein levels for ILK, as well as the downstream targets pAkt and pGSK3β. Suppression of ILK or Akt partially suppressed the enhancement of osteogenic ability induced by periostin overexpression in OVX-BMSCs. Thus, periostin may promote the osteogenic ability of OVX-BMSCs through actions on the ILK/Akt/GSK3β axis.

## Introduction

About 50% of postmenopausal women worldwide suffer from osteoporosis, a common condition of the skeleton (Eastell et al., 2016), with estrogen deficiency being one of its major causes (Friedlander, 2002). Bone is actively remodeled throughout life, which is accomplished by interactions between osteoclasts and osteoblasts. Osteoclasts drive bone resorption and osteoblasts produce new bone matrix. The osteoblast is differentiated from bone marrow stromal cells/skeletal stem cells (BMSCs), which can also differentiate into adipocytes. The switch to adipogenesis or osteogenesis is critical for BMSC in bone regeneration i.e. BMSCs can differentiate into adipocytes at the expense of osteoblasts and vice versa (Gimble et al., 2006). 

Osteoporosis biopsies from humans have revealed the replacement of bone mass by adipose tissue (Meunier et al., 1971). It is well established that the commitment of BMSC to an osteoblast lineage is under tight regulation. A high dose of TGFβ/BMPs is essential for the expression of runt-related gene 2 (Runx2), which is an osteoblast-specific marker (Chen et al., 2012). Furthermore, Hedgehog signaling can interact with BMP signaling to promote osteoblast differentiation (Spinella-Jaegle et al., 2001). Wnt3a (Byun et al., 2014) and Wnt10b (Bennett et al., 2007; Stevens et al., 2010) also promote osteoblast-specific differentiation. On the other hand, Notch signaling has been reported to promote both adipogenic and osteoblast differentiation through disparate mechanisms (Ross et al., 2004; Deng et al., 2008; Song et al., 2015). Several cell line-based studies have revealed that estrogen deficiency impairs osteogenic differentiation. In mice, estrogen receptor-α (ERα) is upregulated by Wnt3a overexpression and thus ER signaling together with Wnt3a can promote osteogenic differentiation (Gao et al., 2013). 

Periostin, a member of the matricellular family, serves a scaffold function for extracellular matrix (ECM) protein assembly and can also bind to cell surface receptors such as integrin and Notch-1. Periostin is not only an organizer of ECM, but also a regulator of cell adhesion and differentiation. Thus, it plays essential roles in several diseases including fibrosis (O’dwyer and Moore, 2017), inflammatory disease (Koh et al., 2019; Ohno et al., 2019; Ryu and Kim, 2020) and tumorigenesis (Ma et al., 2019; Ma *et al.*, 2020), and also regulates bone regeneration (Li et al., 2019). Studies have revealed that ECM proteins and matricellular proteins including Periostin are upregulated after bone injury (Duchamp de Lageneste et al., 2018; Heo et al., 2015). Research on periostin KO mice has suggested a role for periostin in bone development and homeostasis (Norris et al., 2007; Bonnet et al., 2013). In a previous study, Periostin has been shown to participate in osteoblast differentiation through Wnt/β-Catenin signaling (Bonnet *et al.*, 2012; Zhang et al., 2017), which suggests that Periostin may be a novel target for osteoporosis therapy.

To investigate further the role of Periostin in osteogenic differentiation in an estrogen-depleted state, female rats were treated with ovariectomy (OVX) to induce postmenopausal osteoporosis and then BMSCs were isolated. We found that overexpression of periostin could promote osteogenesis of OVX-rBMSCs and this facilitation is likely accomplished through actions on the ILK/Akt/GSK3β axis.

## Material and Methods

### Animals and establishment of the osteoporosis model

All animals were purchased from the laboratory animal center of the PLA Air Force Military Medical University. Animal use and care protocols were conducted according to the Guide for the Welfare Committee of Laboratory Animals of the PLA Air Force Military Medical University (IRB approval number: 20170604). Six-week-old female Sprague-Dawley rats weighting 110 ± 10 g were randomly assigned into OVX (n = 12) or sham groups (n = 12). Rats in the OVX group were subjected to bilateral ovary removal. Rats in the sham group were subjected to a sham operation (i.e., without removal of the ovaries). All the rats were bred for another 3 months before being humanely euthanized with carbon dioxide.

### MicroCT analysis

To identify the establishment of the osteoporotic rat model, specimens were scanned with a microCT system (Siemens, Inveon MicroCT). All images were obtained using Inveon Research Workplace (Siemens).

### Cell isolation, culture and induction of differentiation

Primary BMSCs were isolated from the femur of rats by flushing the bone marrow with DMEM (Gibco) supplemented with 1% FBS (Gibco) and 1% penicillin-streptomycin solution (Gibco). Cells were then incubated at 37°C with added 5% gaseous CO_2_ and 95% humidity. The medium was refreshed every 3 days. After the cells reached 80-90% confluence, they were digested with 0.25% trypsin (Gibco) and passaged at a ratio of 1:3.

For identification of BMSC markers, the third passage of BMSCs was subjected to flow cytometry analysis. Briefly, cells that reached 80% confluence were trypsinized and incubated with CD29 (eBioscience, 11-0291-80), CD34 (Santa Cruz, sc-19587), CD45 (Abcam, ab10558), CD90 (Bio-Rad, MCA47GA), followed by analysis using flow cytometry (CytoFLEX, Beckman Coulter). For each sample, 10,000 events were analyzed.

For osteogenic induction, the third passage of BMSCs were seeded in 6-well plates at a density of 2 × 10^5^ cells/well and cultured in DMEM containing 10% FBS, 10 mM β-glycerophosphate (Sigma), 0.1 μM dexamethasone (Sigma) and 50 mg/L ascorbic acid (Sigma). The medium was refreshed every 3 days.

For adipogenic induction, the third passage of BMSCs were seeded in 6-well plates at a density of 2 × 10^5^/well and cultured in DMEM containing 10% FBS, 10 μM dexamethasone (Sigma), 200 μM isobutylmethylxanthine (Sigma), 0.5 mM IBMX and 10 mg/L insulin (Sigma). The medium was refreshed every 3 days.

### Cell transfection and Akt/GSK-3β signaling modulation

The recombinant lentiviral vector containing periostin was prepared by cloning full length rat periostin cDNA. The PCR primers for periostin cloning were as follows:

(5’ - 3’): Forward: AGGTCGACTCTAGAGGATCCCGCCACCATGGTTCCTCTCCTGCCCTTATC, Reverse: TCCTTGTAGTCCATACCCTGAGAACGGCCTTCTCTTGATCGCCTTCTAGACCCTTGAACCCTTTTGTTG. 

The resulting cDNA was digested with the BamHI/AgeI restriction enzyme and then inserted into the vector GV492 (Genechem). The recombinant lentiviral vector and LentiEasy Packaging Mix (Genechem) were co-transfected into 293T cells using Lipofectamine 2000 (Thermo Fisher) to generate the lentivirus. 

Passage 3 of primary BMSCs were seed into 12-well plates at a density of 2 × 10^5^ cells/well and incubated overnight. Then, the cells were transfected with 10 μL (2.5 × 10^8^ TU/mL) recombinant lentiviral vector or empty vector (multiplicity of infection, MOI = 50). After a 12 h incubation, the medium was replaced with regular medium and incubated for another 48 h followed by subsequent experiments. The transfection efficiency was detected by qPCR.

For inhibition of Akt/GSK-3β in BMSCs overexpressed with periostin, cells were transfected with recombinant lentiviral vector in the presence of 0.5 μM Akt or GSK-3β inhibitor. The ILK inhibitor used was OSU-T315 (MCE, HY-18676) and the Akt inhibitor genistein (MCE, HY-14596). After a 12 h incubation, the medium was replaced with regular medium and incubated for another 48 h and then subsequently experiments were conducted.

### Alkaline phosphatase (ALP), alizarin red and oil red O staining

BMSCs derived from the Sham or OVX groups were seeded on 24-well plates at 1,000 cells/well for 24 h. According to the grouping, cells were untreated or transfected with the corresponding recombinant lentiviral vectors (*vide supra*).

For ALP staining, cells after 7-days induction were fixed in 4% formaldehyde (Sinopharm) for 15 min and then washed 3 times with distilled water. Then the cells were incubated with BCIP/NBT Chromogen Kit (Solarbio) and Nuclear fast red (Sinopharm) according to the manufacturer’s protocol. Quantitative ALP activity was assessed using an alkaline phosphatase assay kit (Beyotime) according to the manufacturer’s protocol. Briefly, cells were lysed with lysis buffer (1% Triton X-100). Optical density was measured using a spectrophotometer (Thermo Fisher Scientific, Multiskan MK3) at 405 nm with p-nitrophenyl phosphate (p-NPP) (Sigma) as the substrate. Relative ALP activity was normalized to the total protein curve.

For alizarin red staining, cells after 21-days induction were fixed in 4% formaldehyde (Sinopharm) for 15 min and then incubated with alizarin red S solution (0.2%, pH 4.2, Solarbio) according to the manufacturer’s protocol. For quantification of the relative degree of mineralization, the deposits were dissolved in 10% hexadecylpyridinium chloride monohydrate (Sigma) solution for 5-10 min. The supernatant was collected and centrifuged at 2,000 rpm for 5 min. Then the absorbance of 100 μL supernatant was measured at 520 nm using a UV spectrophotometer. The results of ARS are expressed as OD_520_ with the mean ± standard deviation.

For oil red O staining, cells after a 14-day induction were fixed in 4% formaldehyde (Sinopharm) and washed with 60% isopropanol. Next, cells were incubated with oil red O (Sigma) followed by staining with Mayer hematoxylin (Sigma) for 1-3 min.

Samples were observed with a phase-contrast microscope (Olympus, BX53) and images captured using cellSens (Olympus).

### RNA extraction and quantitative real-time PCR

Total RNA was isolated with Trizol reagent (Ambion) and reverse-transcribed into cDNA with HiScript® Reverse Transcriptase (VAZYME) according to the manufacturer’s instructions. For real-time PCR, a total of 20 μL of reaction mixture containing 10 μL of SYBR Green Master Mix (VAZYME), 0.4 μL of 50 × ROX Reference Dye 2 (VAZYME), cDNA and primers was incubated in an QuantStudio™ 6 Flex system (ABI). The primers (from 5’ to 3’) are listed in Table 1.

**Table 1 -:** Primer sequences used for real-time PCR.

Gene	Sequence
β-actin F	CACGATGGAGGGGCCGGACTCATC
β-actin R	TAAAGACCTCTATGCCAACACAGT
OCN F	CCTCTCTCTGCTCACTCTGCTG
OCN R	CTATTCACCACCTTACTGCCCTC
BSP F	CAGTTATGGCACCACGACAG
BSP R	CCATGCCCCTTGTAGTAGCT
Runx2 F	TCAGCGTCCTATCAGTTCCC
Runx2 R	ATTCAAAACGGTTGGGGAGC
periostin F	GTTCCTGTGTGACGTTGACC
periostin R	CGGGGCAGCATTCATATAGC

The relative quantification of gene expression was calculated using the 2^−ΔΔCT^ method normalized to β-actin gene expression.

### Western blotting

Total proteins were isolated using RIPA lysis buffer with added PMSF and phosphatase inhibitors (BEYOTIME), and were quantified using a BCA protein assay kit (BEYOTIME). The proteins were separated using 12% SDS-polyacrylamide gel electrophoresis and transferred to PVDF membranes (Millipore). After being blocked with 5% fat-free milk, the membranes were incubated with primary antibodies overnight at 4 °C. The primary antibodies used were rabbit anti-Periostin (Abcam, ab14041, 1:1000), mouse anti-β-actin (BOSTER, BM0627, 1:200), rabbit anti-ILK (Abcam, ab76468, 1:1,000), rabbit anti-pAkt (CST, 4060, 1:2,000), rabbit anti-Akt (CST, 4691, 1:1,000), rabbit anti-pGSK3β (Abcam, ab68476, 1:1,000), rabbit anti-GSK3β (Proteintech Group, Inc., 22104-1-AP, 1:1,000). Then, the membranes were incubated with HRP-conjugated goat anti-mouse IgG (BOSTER, BA1051, 1:50,000) or HRP-conjugated goat anti-rabbit IgG (BOSTER, BA1054, 1:50,000) for 2 h at 37°C. The blots were visualized with Pierce™ ECL western blotting substrate (Thermo, NCI5079) and then exposed to X-ray films (Kodak, XBT-1). The relative protein levels were quantified using Image J software (NIH, Bethesda, MD, USA).

### Statistical analysis

All experiments were repeated at least 3 times. Data were recorded in Excel (Microsoft) and analyzed using GraphPad Prism 7 (GraphPad Software, La Jolla, USA). All data are presented as the mean **±** SD. An unpaired *t*-test was used to analyze the difference between 2 groups and two-way ANOVA was used to identify differences between 3 or more groups.

## Results

### Characterization of the OVX model

**Figure 1 -:**
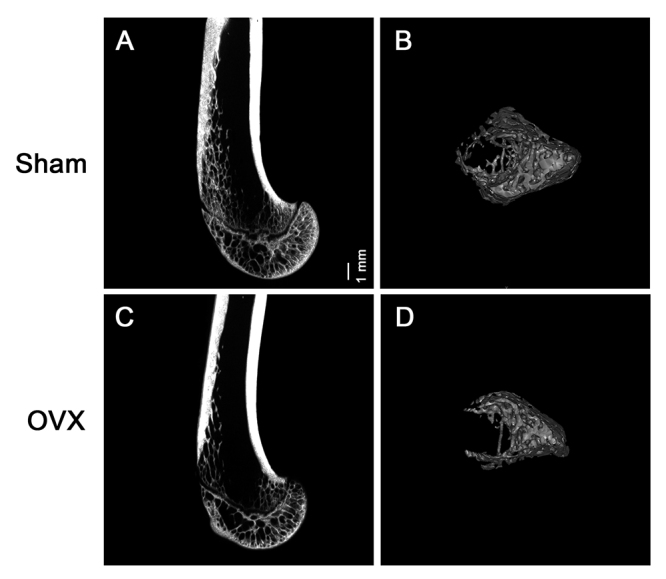
Ovariectomy leads to osteoporosis in rats. (A-D) Representative MicroCT images of distal femurs. (A) and (B) are from rats in the sham group. (C) and (D) are from rats in the OVX group. Scale bar = 1 mm.

**Figure 2 -:**
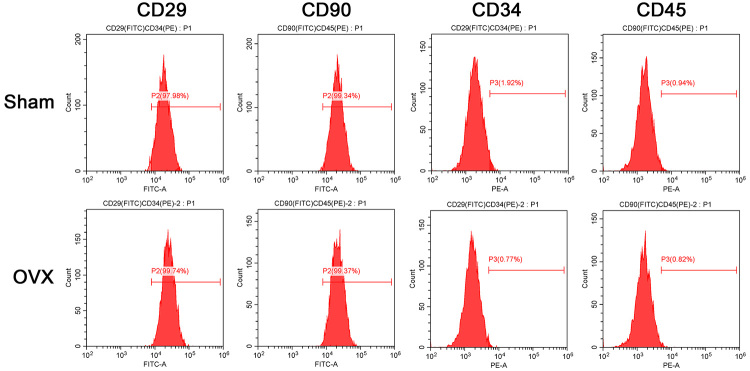
Identification of BMSCs by flow cytometry analysis. Representative images of flow cytometry analysis. Sham-BMSCs were positive for the surface antigens CD29 (97.98%) and CD90 (99.34%) and negative for CD34 (1.92%) and CD45 (0.94%). OVX-BMSCs were positive for the surface antigens CD29 (97.74%) and CD90 (99.37%) and negative for CD34 (0.77%) and CD45 (0.82%).

Three months after establishment of the OVX model, MicroCT was used to quantify the microstructural parameters of the femurs in both groups. The results revealed that the distal femur in OVX was thinner than in the sham group (Figure 1A-D), which indicated the successful establishment of the osteoporotic rat model. Therefore, we isolated rBMSCs from both the sham and OVX groups. Flow cytometry analysis revealed that the cells were CD90+ CD29+ and CD34- CD45- (Figure 2), which confirmed that the isolated cells were BMSCs.

### Characterization of rBMSCs after *in vitro* culture

**Figure 3 -:**
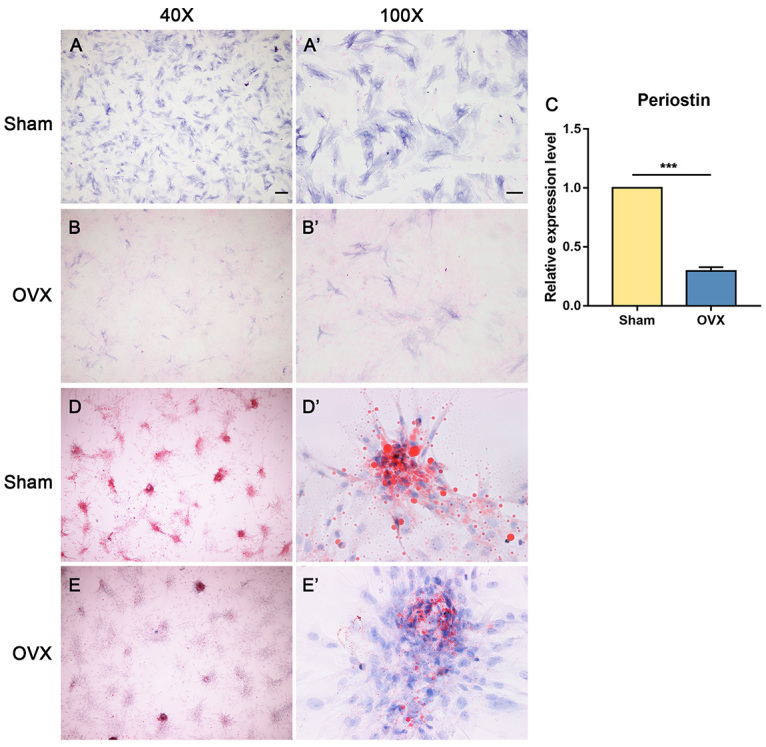
BMSCs derived from OVX rats exhibited impaired osteogenesis ability, adipogenesis ability and reduced expression of periostin. (A-B’) Representative images of ALP staining on day 7 after osteogenesis induction. (A, A’) Sham-BMSCs express ALP, while (B, B’) OVX-BMSCs express less ALP. (C) Graph shows the quantification of the relative expression level of periostin on day 7 after osteogenesis induction. Data are presented as the mean ± SD. ^***^ *P* < 0.001. (D-E’) Representative images of oil red O staining on day 14 after adipogenesis induction. (D, D’) Sham-BMSCs exhibited intracellular lipid droplets. (E, E’) OVX-BMSCs exhibited less lipid droplets. The images are from 1 of 3 independent experiments. (A, B, D, E) Magnification × 40, scale bar = 100 μm. (A’, B’, D’, E’) Magnification × 100, scale bar = 200 μm.

The BMSCs from both the sham and OVX groups were cultured through passage 10. After primary seeding, passage 3 rBMSCs were employed for osteogenic induction. After being induced for 7 days, rBMSCs were stained with ALP, an early osteogenic differentiation marker, to check cell fates. The rBMSC derived from the sham group (sham-rBMSC hereinafter) exhibited intensive ALP staining 7 days after induction (Figure 3A, A’). However, the rBMSC derived from the OVX group (OVX-rBMSC hereinafter) secreted less ALP (Figure 3B, B’), which indicated that osteoblastic differentiation was significantly inhibited. The expression of periostin was significantly reduced in OVX-rBMSC compared to sham-rBMSC (Figure 3C). In contrast, primary rBMSCs were also induced for adipogenic differentiation. After a 14-day induction, the sham-rBMSC (Figure 3D, D’) exhibited a much greater increased intensity of oil red O staining compared to OVX-rBMSC (Figure 3E, E’). Thus, adipogenic differentiation was significantly impaired after OVX treatment.

### Periostin promotes rBMSCs osteoblastic differentiation

**Figure 4 -:**
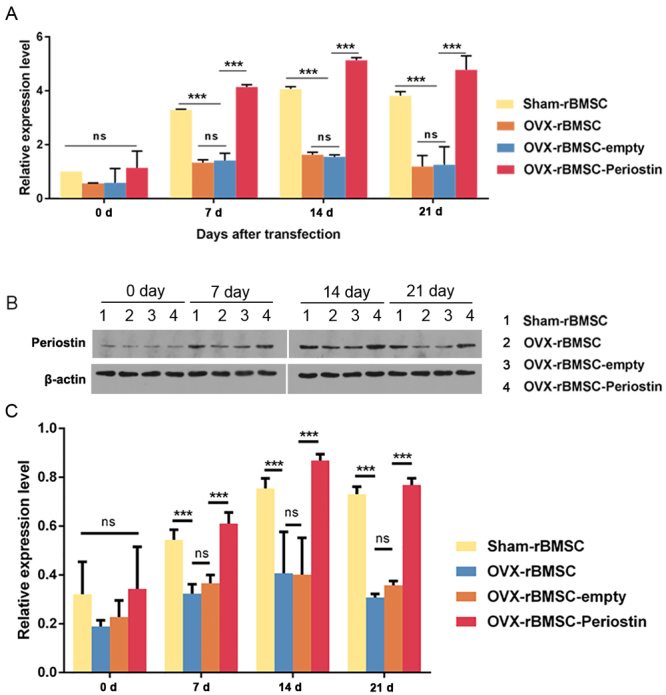
Overexpression of periostin elevated periostin mRNA and protein levels after osteogenesis induction. (A) The graph shows the quantification of the relative expression levels of periostin in the indicated groups on day 0, day 7, day 14 and day 21 after osteogenesis induction. (B) The expression levels of periostin in the indicated groups on day 0, day 7, day 14 and day 21 after osteogenesis induction were investigated using western blotting; β-actin served as the reference gene. The image is representative of 3 independent experiments. (C) Quantitative western blotting was performed using Image J software. Data are presented as the mean ± SD of 3 independent experiments. ns, not significant. ^**^ *P* < 0.001; One-way ANOVA.

Since we found a reduction in Periostin expression in rBMSCs derived from the osteoporosis model rats, we investigated its effects on rBMSC osteogenic differentiation. To overexpress periostin in rBMSC, lentivirus-mediated transfection was employed. 48 h after lentivirus transfection, the osteogenic induction medium was used for rBMSC culture. First, the transfection efficiency was evaluated on day 0, day 7, day 14 and day 21 after transfection by qRT-PCR. Total RNA was isolated and the transcription level of periostin was determined. On day 0, there was no significant difference in the periostin mRNA level among the 4 groups. However, on day 7, day 14 and day 21, periostin mRNA levels were reduced in OVX-rBMSC and OVX-rBMSC transfected with empty vector (OVX-rBMSC-empty), compared to sham-rBMSC. rBMSC-OVX transfected with periostin (OVX-rBMSC-periostin) significantly restored the periostin mRNA expression level (Figure 4A); the protein level of Periostin was determined using western blotting. In sham-rBMSC, the Periostin protein level was increased over time. In addition, Periostin protein was upregulated in OVX-rBMSC-periostin at each time point studied compared to OVX-rBMSC or OVX-rBMSC-empty. More importantly, as the osteogenic induction progressed, Periostin protein levels also increased in OVX-rBMSC-periostin (Figure 4B, C). These results indicated that the expression of Periostin could be induced after osteogenic induction in sham-rBMSC. Furthermore, lentivirus-mediated periostin overexpression could restore its expression, which was suppressed in OVX-rBMSC.

**Figure 5 -:**
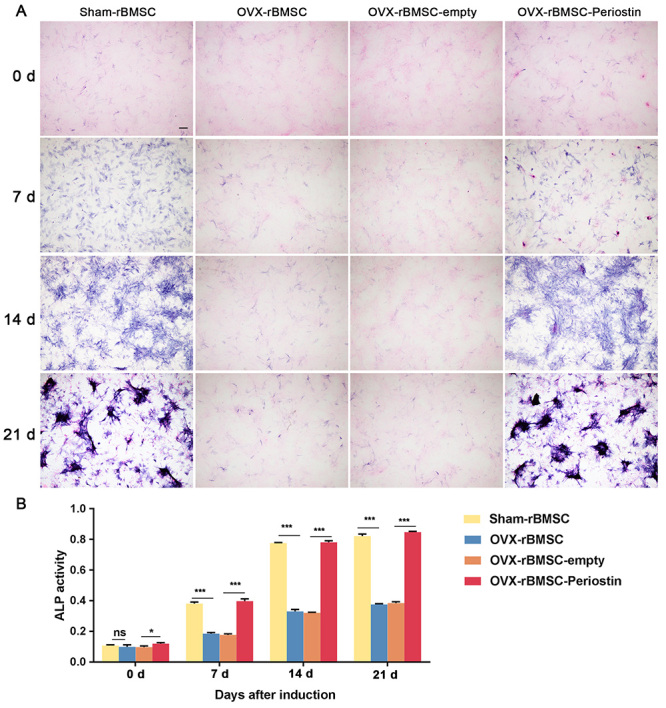
Overexpression of periostin promotes ALP production by OVX-BMSCs. (A) Representative images of ALP staining of the indicated groups on day 0, day 7, day 14 and day 21 after osteogenesis induction. The images are from 1 of 3 independent experiments; Magnification × 40, scale bar = 200 μm. (B) Graph shows quantitative analyses of ALP activity of the indicated groups on day 0, day 7, day 14 and day 21 after osteogenesis induction. Data are presented as the mean ± SD of 3 independent experiments. ns, not significant. ^*^ *P* < 0.05, ^***^ *P* < 0.001; One-way ANOVA.

**Figure 6 -:**
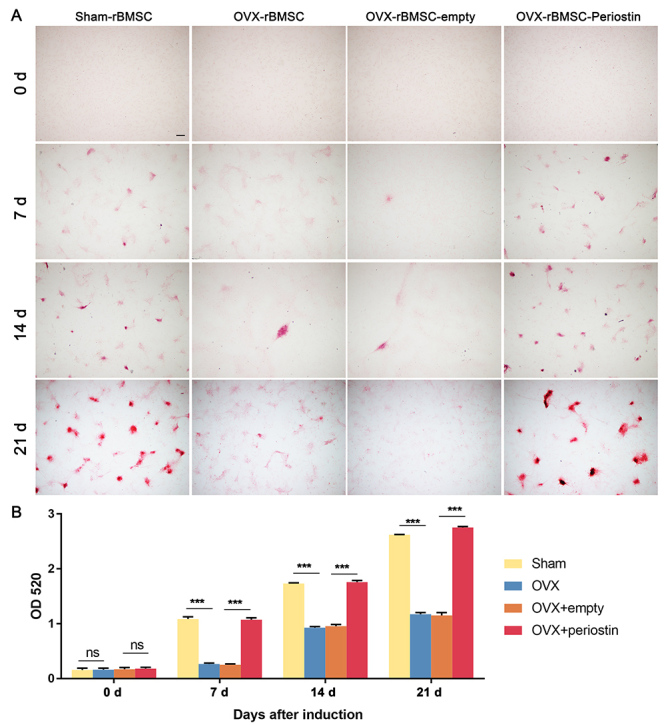
Overexpression of periostin increased ARS staining in OVX-BMSCs. (A) Representative images of ARS staining of the indicated groups on day 0, day 7, day 14 and day 21 after osteogenesis induction. The images are from 1 of 3 independent experiments, magnification × 40, scale bar = 200 μm. (B) Graph showing quantitative analyses of ARS of the indicated groups on day 0, day 7, day 14 and day 21 after osteogenesis induction. Data are presented as the mean ± SD of 3 independent experiments. ns, not significant. ^***^ *P* < 0.001; One-way ANOVA.

In order to assess the osteogenic differentiation ability of OVX-rBMSC-periostin, ALP staining was employed to evaluate the fate of cells. After being transfected with corresponding lentivirus vectors or untreated, cells of the different groups underwent osteogenic induction. ALP staining was assessed 7 days post induction. Overexpression of periostin significantly restored ALP activity and calcium mineralization formation in rBMSC-OVX (Figure 5A, B). Alizarin red S staining was used to monitor calcium deposit formation after a 21-day induction. Overexpression of periostin remarkably increased calcium nodule formation in rBMSC-OVX compared to the controls (Figure 6A, B). These results indicated that Periostin could promote osteoblastic differentiation *in vitro*.

### Periostin regulates rBMSCs osteoblastic differentiation through the ILK/Akt/GSK3β pathway

**Figure 7 -:**
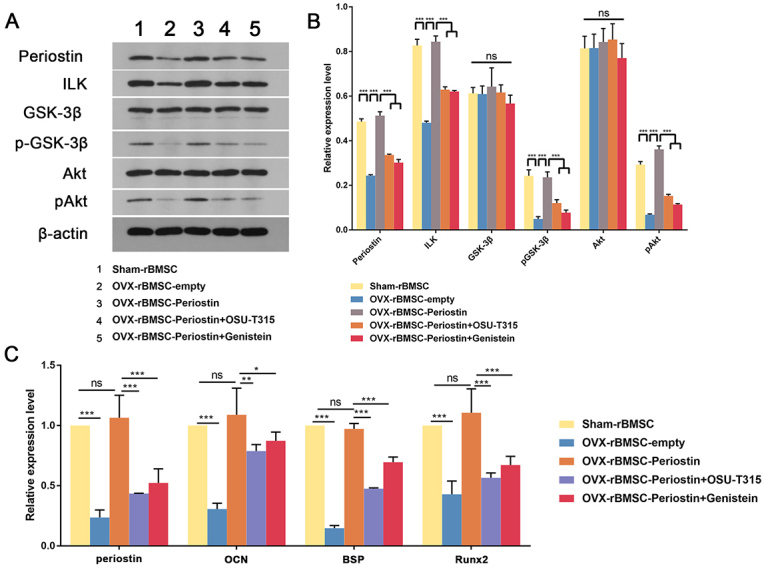
Periostin regulates the expression of ILK, pAkt and GSK3β in OVX-BMSCs. (A) The expression levels of periostin, ILK, GSK3β, pGSK3β, Akt, pAkt in the indicated groups on day 7 after osteogenesis induction were examined by western blotting; β-actin served as the reference gene. (B) Quantitation of western blotting in (A) was performed using Image J software. (C) Graphs showing the relative mRNA expression levels of periostin, OCN, BSP and Runx2 in the indicated groups on day 7 after osteogenesis induction. Data are presented as the mean ± SD of 3 independent experiments. ns, not significant. ^*^ *P* < 0.05, ^**^ *P* < 0.01, ^***^ *P* < 0.001; One-way ANOVA.

**Figure 8 -:**
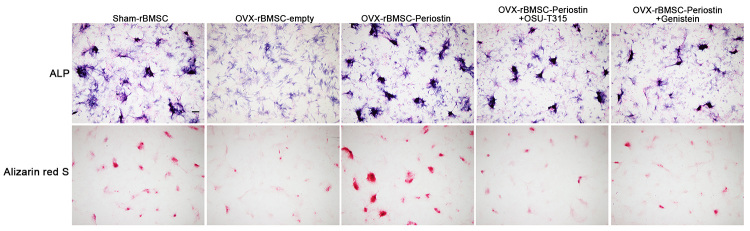
Periostin promotes the osteogenesis ability of OVX-BMSCs in part by action on the ILK/Akt/GSK3 axis. (A) Representative images of ALP staining of the indicated groups on day 7 after osteogenesis induction. The images are from 1 of 3 independent experiments, magnification × 40. (B) Representative images of ARS staining of the indicated groups on day 7 after osteogenesis induction, magnification × 40, scale bar = 200 μm.

It has been reported that ILK-deficient MC3T3-E1 cells exhibit a stronger mineralization ability (El-Hoss et al., 2014), indicating that ILK may slow down osteoblast maturation. On the other hand, Akt/GSK3β signaling also promotes osteoblast differentiation. Thus, we first evaluated ILK, Akt and GSK3β protein levels in the controls and OVX-rBMSC-periostin using western blotting. Of note, ILK was significantly reduced in OVX-rBMSC in comparison to sham-rBMSC. However, overexpression of periostin could restore ILK protein levels. Neither Akt nor GSK3β protein levels were altered in each group. Nonetheless, the phosphorylation of Akt and GSK3β were attenuated in OVX-rBMSC, as detected by antibodies against pAKT and pGSK3β and these phosphorylated proteins were relieved upon transfection with periostin in OVX-rBMSC (Figure 7A, rows 1-3). These results indicated that Periostin could regulate the activity of the ILK/Akt/GSK3β pathway. To elucidate whether Periostin regulates rBMSCs osteoblastic differentiation by action on the ILK/Akt/GSK3β pathway, rBMSC-OVX transfected with periostin were treated with the ILK inhibitor, OSU-T315 or the Akt inhibitor, genistein, before being stained with ALP to evaluate the fate of cells. First, we found that ILK and pAkt protein levels were significantly reduced after treatment with inhibitors, and subsequently that the level of pGSK3β was also reduced (Figure 7A, rows 3-5, B). Furthermore, the results of ALP and alizarin red S staining revealed that both inhibitors could reduce the osteoblastic differentiation of periostin-OVX-rBMSC (Figure 8). Consistent with the above data, the qPCR results revealed reduced expressions of OCN, BSP and Runx2 after inhibitor treatment (Figure 7C). Interestingly, periostin was also downregulated at both the mRNA and protein level after ILK/Akt/GSK3β activity was blocked in periostin-OVX-rBMSC. These results indicated that Periostin was also regulated by ILK/Akt/GSK3β signaling activity.

## Discussion

In bone, Periostin is a key ECM component of the periosteum. Moreover, it is known to promote chondrocyte differentiation by enhancing Akt phosphorylation via integrin as demonstrated in a 3D culture model (Inaki et al., 2018). In the present study, we found that Periostin was reduced in BMSCs derived from the osteoporosis rat model and that overexpression of periostin could enhance the osteogenic ability of BMSCs, in part by activating the ILK/Akt/GSK3β axis. In line with the expression level of Periostin, ILK, pAkt and pGSK3β levels were also reduced in BMSCs derived from female rats with osteoporosis. However, all of these three proteins recovered to the level equivalent to the control group after periostin overexpression. It is also worth noting that both mRNA and protein levels of Periostin were reduced when the ILK/Akt/GSK3β axis activity was suppressed, although periostin was simultaneously overexpressed by lentivirus. Thus, it is reasonable to conclude that the stability of periostin mRNA or Periostin protein was affected by the suppression of Akt/GSK3β signaling. Since Periostin expression was not completely abolished in BMSCs-OVX, we cannot rule out the possibility that the constitutive expression of Periostin was attenuated when the ILK/Akt/GSK3β axis was blocked. These results suggest a negative feedback regulation of Periostin activity in osteogenesis and that the underlying mechanisms involved require further investigation. 

Osteoblast differentiation is tightly regulated by various soluble factors including systemic and local factors, which include the sex steroids and extracellular signals. In addition, mechanical stress is also essential for osteoblast differentiation. Long-term mechanical unloading, such as prolonged bed rest, immobilization or microgravity in space, can inhibit osteoblast differentiation (Jaworski et al., 1980), because mechanical stress is critical for osteoblast differentiation. Integrin is known for its ability to relay the mechanical cue from the environment to the nucleus through stress fiber formation (Burridge and Guilluy, 2016). More importantly, mechanical stress promotes osteogenic MC3T3-E1 cell activity via integrin-β1-mediated β-catenin signaling (Yan et al., 2016). After binding to integrin, ILK has been reported to participate in the integrin-ERK1/2 cascade in the process of periodic mechanical stress-induced chondrocyte proliferation and matrix synthesis. It is noteworthy that the expression of ILK is also induced by mechanical stress (Song et al., 2016). In contrast, ILK has been reported to be involved in the phosphorylation of Akt and GSK3β (Liu et al., 2017). In our study, ILK, pAkt and pGSK3β were upregulated by Periostin. In addition, the levels of pAkt and pGSK3β were altered by modulation of Periostin, rather than AKT and GSK3β. Thus, in the scenario of osteoblast differentiation, Periostin may regulate osteoblast differentiation through the mechanical transduction-based ILK/pAkt/pGSK3β axis.

The ILK/Akt/GSK3β axis has been reported to play roles in cancer cell growth and survival (Liu et al., 2017). Our results suggest a potential relationship between Periostin and ILK/Akt/GSK3β in the process of BMSC differentiation. As an upstream regulator of GSK3β, canonical Wnt/β-catenin signaling plays a role in osteogenic differentiation (Kikuchi et al., 2007; Maupin et al., 2013). Similarity, in human ankylosing spondylitis, a chronic inflammatory disease, Periostin is downregulated and associated with the Wnt pathway (Solmaz et al., 2018). In CTLA4-modified MSCs, Periostin promoted osteogenic differentiation via the Wnt signaling pathway. Our study has provided molecular evidence that Periostin plays a key role in osteogenic differentiation via Wnt signaling in bone cells. 

Since Periostin is a component of ECM proteins, it is able to communicate with the extracellular matrices. For example, in hematopoietic stem cells, Periostin can interact with Notch1 via CCN3 to maintain the hematopoietic stem cells (HSCs) or progenitor cells. More importantly, Notch pathway molecules, including Notch1, have been found to be increased in BMSCs derived from ovariectomized mice, which might participate in the prevention of osteogenic differentiation. Therefore, it is unlikely that Periostin promotes BMSC differentiation through the Notch pathway. Another extracellular matrix protein, bone morphogenetic protein-1 (BMP1), is also supported by Periostin activity. The interaction between BMP1/LOX1 and Periostin promotes collagen cross-linking, which may subsequently enhance TGF signaling activity. However, when human mesenchymal stem cells were incubated with BMP1/LOX1-modified cell matrices, the adipogenic differentiation ability of MSC was enhanced (Rosell-Garcia and Rodriguez-Pascual, 2018). Another study reported that upregulation of BMP1 promoted osteogenic differentiation (Zhang et al., 2019). Thus, whether Periostin can promote BMSC osteogenic ability through BMP1 and TGF pathways remains to be unequivocally established. 

Previous studies have demonstrated that periostin can be regulated by estrogen. In human periodontal ligament cells, estrogen stimulation significantly increased periostin transcription. Our previous study also revealed that 17β-E2 upregulated periostin expression in ovariectomized rats. Estrogen plays a protective role in bone formation and homeostasis; thus, it is also used as a drug to reduce bone loss. To date, most research on bone formation has concentrated on the characterization of BMSCs. whereas there is a paucity of studies that used BMSCs derived from postmenopausal osteoporosis model rats. First, the rodent postmenopausal osteoporosis model established by ovariectomy only reflects acute effects of estrogen deprivation. Although the osteogenic ability of BMSCs derived from OVX-rats is impaired in several passages *in vitro*, it is no longer regulated by systemic estrogen. Thus, we cannot conclude that the promotion of osteogenic ability by Periostin is under the control of estrogen. Second, it has been hypothesized that stem cells from the periosteum and bone marrow play distinct roles and bone modeling and remodeling benefit more from stem cells in the periosteum than those in the bone marrow (Duchamp de Lageneste et al., 2018). Third, specialized niche, which could not be mimicked in cell line-based studies, are essential for proliferation, survival and differentiation of skeleton stem cells. Altogether, more *in vivo* and *in vitro* studies are necessary to provide new insights into the molecular pathology of Periostin-related postmenopausal osteoporosis.

In summary, our findings have shown that Periostin can promote BMSC osteogenic differentiation through the ILK/pAkt/pGSK3β axis in BMSCs derived from OVX-rats. Our findings suggest that Periostin may serve as a therapeutic target of osteoporosis. In subsequent research, *in vivo* Periostin knock-out experiments will be needed to confirm further its importance in the osteogenic effect in postmenopausal osteoporosis rat models.
